# Aneurismal Tumour of Bone

**Published:** 1845-06

**Authors:** John Topham


*Aneurismal Tumour of Bone.* By John Topham, Esq. M. P.—A young man, by trade a marble-cutter, was admitted into the Hôpital des Cliniques, Paris, March 3, 1845, under the care of M. Nélaton. The history given by the patient of the complaint under which he labours is as follows:—

Three months ago he became aware for the first time of the existence of an enlargement in the vicinity of the right knee-joint. This commenced on the inner side of the limb, just above the line of flexion of the joint. It was unattended by pain, and the patient has continued to walk about up to the present time, the tumour continuing gradually to increase in size, but not occasioning any inconvenience excepting during the act of walking down a very steep place, such as a flight of stairs, when slight uneasy sensations are experienced in it.

*Present Condition.*—There is now an enlargement occupying the situation of the internal condyle of the right femur. The dimensions of this tumour are six centimetres in the transverse direction, and about eight centimetres as measured from before backwards. The exact limits to which the disease extends are difficult to define, as at

35*

its upper portion only is there a salient edge perceptible. The swelling occupies much more of the anterior than of the posterior portion of the limb.

The integuments covering the tumour are marked by numerous vascular patches, which are probably effects of the previous treatment to which the patient has been subjected, vesicatories, &c. having been applied, with the hope of causing absorption of the diseased structure.

The general surface of the tumour offers a hard resisting surface to the hand of the surgeon, it being evidently formed of an osseous shell; but over the internal condyle of the femur, precisely in the situation in which the patient describes the disease as having commenced, there is a point which offers a less degree of resistance to pressure, and in which the bony tissue appears to have become absorbed. This point is the only one in which pressure occasions pain to the patient, the rest of the surface of the tumour possessing no preternatural degree of sensibility. No pain is complained of in the thigh or leg, nor does pressure made over the course of the crural vessels occasion any abnormal sensation.

On placing the hand upon the tumour, a distinct pulsation is perceived, synchronous with each beat of the femoral artery. The swelling at the same time is felt to increase in size, especially in that situation in which there is naturally the least resistance.

Pressure made upon the femoral artery occasions the immediate cessation of this pulsatory motion in the tumour, and this occurs equally, whether the pressure be made in the upper or in the middle third of the thigh. Coincidently with the stoppage of the pulsation, the tumour is felt to become soft, and to sink down to a certain extent under the hand in the situation in which there is least resistance in the parietes.

On applying the ear to the swelling, (the femoral artery being free,) a slight sound is heard synchronous with the arterial pulsations, but this is in no way comparable with the bruit heard in an ordinary aneurism, and is so slight as to be with difficulty distinguishable.

M. Nélaton determined upon placing a ligature upon the femoral artery, in the hope of thus arresting the progress of the tumour. The vessel was accordingly tied in the middle portion of its course, this situation being selected because it had been previously found that pressure made upon this part of the artery commanded the flow of blood into the tumour as completely as when excited upon the vessel in the upper third of the thigh; and because, in case of secondary hæmorrhage, the operation of Scarpa would still remain practicable. The operation was performed on the 10th of March.

The immediate result of this procedure was to cause the disappearance of all pulsation in the morbid structure, but at the end of seven days this phenomenon again made its appearance, so that the result of the operation has been unsuccessful.

I may remark, in conclusion, that in the case just described are combined all the characters mentioned by authors as appertaining to this peculiar affection, with the exception of *one.*

M. Lallemand, of Montpellier, describes a sensation of *crackling* to be perceptible to the finger when pressure is made upon the tumour. This is due to the breaking down of minute osseous septa which exist in the substance of the morbid mass. The noise produced has been compared to the "crumpling" of parchment. The above phenomenon also presented itself in a case treated by M. Roux. In the present instance, however, nothing of this nature was perceptible either to the surgeon or to the patient.—*London Lancet.*

